# A racial equity approach to nonprofit hospitals’ community benefit programs: an exploratory study

**DOI:** 10.3389/fpubh.2025.1514085

**Published:** 2025-05-14

**Authors:** Cherie Conley, Crytstal N. Lewis, Simone Rauscher Singh

**Affiliations:** ^1^School of Nursing, University of Michigan, Ann Arbor, MI, United States; ^2^The Ohio State University, Columbus, OH, United States; ^3^School of Public Health, University of Michigan, Ann Arbor, MI, United States

**Keywords:** nonprofit hospitals, community benefit, equity, racial equity, health policy

## Abstract

**Introduction:**

The aim of this study was to explore the factors and processes that drive nonprofit hospitals’ willingness and ability to implement equity-focused community benefit initiatives – specifically initiatives aimed at addressing social and structural determinants of health and inequities associated with racism.

**Methods:**

We conducted a cross-sectional qualitative study using semi structured interviews guided by the EquIR Implementation science framework and Browne’s racial equity approach to community benefit program implementation. Browne’s racial equity approach includes five strategies: (1) prioritizing community building strategies in communities impacted most by racism, (2) allocating resources to address structural determinants that reflect racism, (3) providing leadership and employment opportunities for community members and organizations impacted by racism, (4) addressing and considering multiple intersecting identities, and (5) removing organizational barriers to allocating resources to address health inequities along racial lines.

**Results:**

We conducted 24 interviews with leaders of 23 hospitals and health systems representing the Northeast, South, Midwest and West United States regions. We used directed content analysis to analyze interview data. The racial equity strategies most often used were 1, 2, and 3. The strategies least likely to be mentioned were 4 and 5.Two of the 23 health systems engaged in all five strategies.

**Conclusion:**

Health systems have the potential, partnerships, and resources to incorporate a racial equity lens into the planning, design, and implementation of their community-based initiatives. Utilizing a racial equity approach that includes prioritizing affected communities and providing resources and strategies to overcome barriers to accessing those resources is a template that can be used by hospitals to get closer to more effectively achieving this goal.

## Introduction

The 2002 Institute of Medicine report “Unequal Treatment” detailed the stark inequities in health care and health outcomes experienced by racial and ethnic minorities (1). Health inequities limit the ability of racial and ethnic minorities in the US to attain optimum health and cost over 200 billion dollars annually in unnecessary health expenditures and lost productivity (2). Reducing inequities in health and healthcare requires a concerted effort by policymakers, practitioners, and researchers at the local, state, and national levels. Members of racial and ethnic minorities who often have less access to quality health care while also being more exposed to social and environmental hazards are most at risk for poor health. Optimally, racial equity in health in the US would result in decreased disparities in health outcomes such as life expectancy and chronic conditions such as hypertension, high cholesterol, diabetes, asthma, and cancer (2). The purpose of this study was to explore whether or not and how nonprofit hospitals address racial equity in the planning, design, and implementation of initiatives promoting health equity and targeting social and structural determinants of health.

Nonprofit hospitals, which represent almost 60 percent of community hospitals in the United States, receive an estimated 24 billion dollars annually in tax benefits (3). In return, the Internal Revenue Service (IRS) mandates that these hospitals provide community benefit (CB), (i.e., services aimed at improving the health of the communities they serve) (4). While the majority of CB spending goes toward uncompensated care, hospitals also invest in broader community health improvement efforts. Prior analysis of the community health initiatives of a representative 20% sample of US nonprofit hospitals found that almost 75% of hospitals used health equity as a guiding theme when developing their IRS-mandated community health needs assessment (CHNA) (5). However, to date, it is unclear how hospitals have leveraged their community benefit programs to address social and structural determinants of health and health equity, specifically prioritizing structural racism (6–8).

Prior work has shown that while hospitals are engaged in equity work there often is not explicit focus on racial equity and structural racism. For instance, Doherty et al. (9) interviewed healthcare experts and executives to identify factors associated with systematic organizational change to promote equity. While approaches varied based on individual location and context, six factors were most often cited as essential to addressing equity - (a) committed and engaged leadership; (b) integrated organizational structure; (c) commitment to quality improvement and patient safety; (d) ongoing training and education; (e) effective data collection and analytics; and (f) stakeholder communication, engagement, and collaboration. In this study, important information was gained about how organizational change can be carried out to promote equity; however, race and the potential connection between equity, structural determinants of health and race was rarely mentioned outside of demographic data collection. For example, race neutral initiatives can ignore the structural barriers embedded in social systems, laws, and health care that have historically disadvantaged racial and ethnic minorities’ ability to improve or maintain good health. In so doing, they can serve to reinforce systems of disadvantage instead of improving equity (10).

To explore more specifically how hospitals might address equity in vulnerable communities, Rozier et al. (11) surveyed over 200 executives from nonprofit hospitals, local health departments, and community organizations. They found that executives were less focused on creating programs that prioritized vulnerable populations or committing to long-term investments in communities – two factors that are tied directly to racial equity and structural determinants of health. Similarly, Castaneda et al. (12) interviewed a combination of leaders from health departments, health systems, academic institutions and community-based organizations to examine how cross sectoral partnerships are used to address and improve racial health equity initiatives in four different US cities. They also found that while equity is a focus, more work and attention is needed to specifically address root causes of racial inequity that lead to disparate health outcomes.

## Materials and methods

### Conceptual model

**Figure 1:**
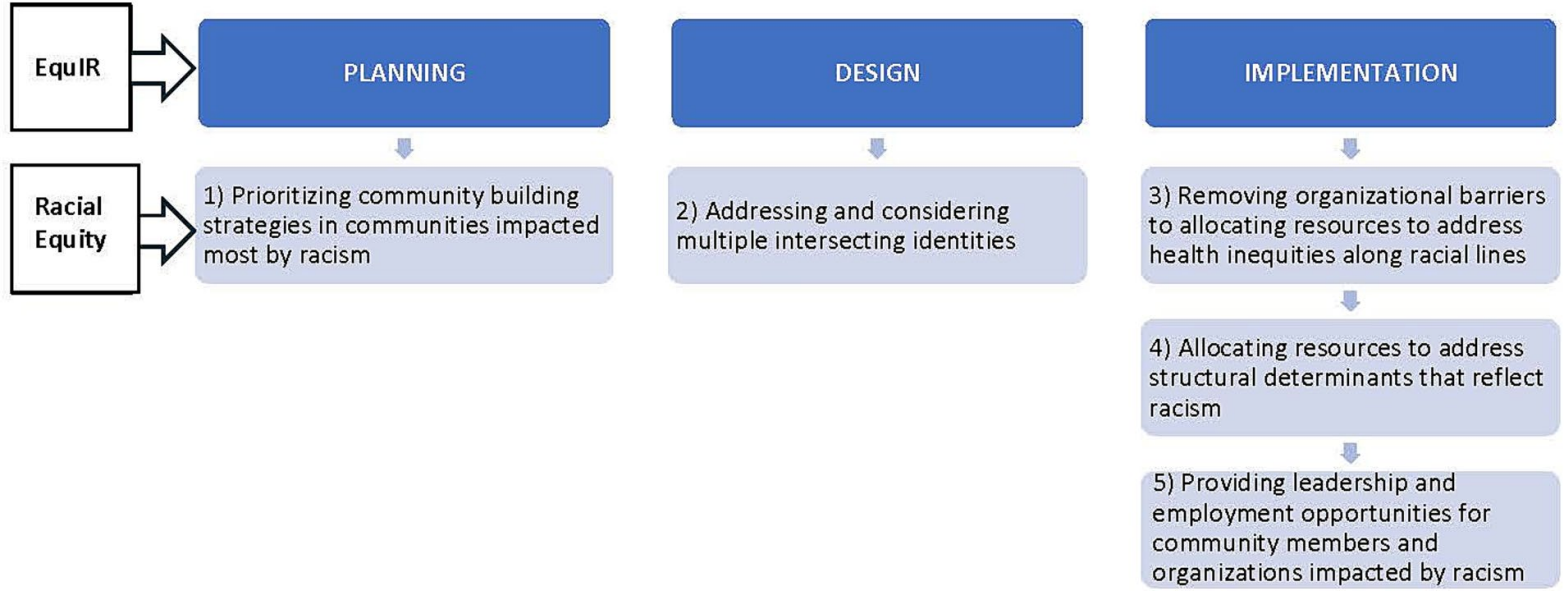
A racial equity approach to program planning, design, and implementation.

The proposed project is guided by Browne’s racial equity approach to community benefit program implementation (13) and the Conceptual framework of equity-focused implementation research for health (EquIR) (14) (Figure 1). Browne’s approach is based on a combination of published paradigms from the field of sociology and economics and includes five organizational strategies to include a racial equity lens when designing community benefit initiatives: (1) prioritizing community building strategies in communities impacted most by racism, (2) allocating resources to address structural determinants that reflect racism, (3) providing leadership and employment opportunities for community members and organizations impacted by racism, (4) addressing and considering multiple intersecting identities, and (5) removing organizational barriers to allocating resources to address health inequities along racial lines.

For this study, we embed the racial equity strategies within the major steps of EquIR, an implementation science framework which provides concrete steps to guide organizational and community collaborations to implement equity focused programs. EquIR is based on other well-known implementation science frameworks (i.e., consolidated framework for advancing implementation science, CFIR) (15), used to guide program implementation. The four steps of EquIR are planning, designing, implementation, and evaluation. Within each step are subcategories that provide more specific directions that also represent Browne’s racial equity approach. More specifically, planning includes identifying disadvantaged groups, quantifying health inequities, and suggesting initiatives to address inequities. Designing includes identifying barriers, facilitators and key factors for implementing equity initiatives. Implementation includes developing a communication strategy, defining incentives and strategies to overcome barriers, and strategies for monitoring and evaluation. Interview questions were guided by the first three steps of the EQuIR framework – planning, design, and implementation - which capture all five of the strategies in Browne’s racial equity approach. Evaluation was not included because many health systems are in their initial stages of planning and implementation and have not yet evaluated long-term effects or scaled their programs.

### Setting and sample

The study was conducted using the directed content analysis approach to qualitative research outlined by Kibiswa (16) and the Consolidated Criteria for Reporting Qualitative Research (COREQ) (Supplementary material) (17). Directed content analysis is a deductive analytical coding approach driven by frameworks identified *a priori* (16). For this study, the a priori frameworks used were EquIR and Browne’s racial equity approach to community benefit program implementation. The study was exempt from the university’s Internal Review Board (IRB).

The research team used purposive and convenience sampling of nonprofit hospitals across the United States for this study. Hospitals and hospital administrators were recruited in three ways. First, two study team members who in the past worked with hospital community benefit managers and with the Catholic Health Association (whose mission includes providing for the poor and underserved), reached out to their contacts via email to recruit potential interviewees. Each team member identified systems and potential interviewees they were familiar with and whose contact information they had. Second, the study team recruited participants while attending the American Hospital Association’s (AHA) 2023 conference on health equity (18). Third, the study team reached out to the AHA nursing board director via email and requested assistance inviting members involved in community benefits to participate in this study.

The sampling frame was chosen in order to connect with health systems that are likely to address SDOH and/or equity. While some systems may develop comprehensive publicly facing reports that reflect their efforts, others, for various reasons such as staffing constraints, or actively designing their strategic approach, may not - even though they are in fact growing their efforts to address equity. The purpose of the interviews was to explore more deeply through interviews with representative hospital key informants, whether or not and how health systems address racial equity. Including hospitals intentionally addressing equity and SDOH allowed the study team to note variations in addressing racial equity even among a sample likely endeavoring to address equity overall.

Finally, to capture maximum variation in responses, the study team aimed to contact hospitals from four regions of the US (West, Midwest, South, Northeast), teaching and non-teaching, rural and non-rural, higher and lower bed counts, and from states with and without community benefit reporting requirements.

### Data collection

We completed semi-structured interviews from October 2022 – July 2023. All interviews were conducted via Zoom and recorded with participants’ consent. Only the invited hospital representatives and study team members were present for the interviews. In a qualitative grounded theory study of hospital community administrators, saturation was reached after interviewing 38 hospital community leaders (11). Therefore, the goal of this study was to interview between 15 and 38 administrators. Interviews began with an open-ended question ‘Tell me about your role and how long you have been with your organization’ to allow the interviewee to provide an overview of their hospital and their duties with the hospital. Next, interviewees were asked about their experience initiating and implementing equity focused community benefit initiatives at their organization. Subsequent questions were guided by probes specific to the three phases of equity focused implementation outlined in EQuIR - design, planning, and implementation (i.e., ‘Can you share some examples of equity focused programs and walk me through the process of planning, designing, implementing the program’, ‘What were the major facilitators and challenges to your hospital implementing equity focused programs’). Interview questions such as ‘Did you use a framework to guide your work?’ and ‘What type of data was used to support and make the case for equity initiatives?’, allowed the study team to get a comprehensive understanding of the organization’s perceptions of equity and to find out what, if any strategies used support racial equity. Two members of the study team conducted interviews to allow for debriefing post-interview to discuss major themes and to note any new ideas presented by the interviewee. Interviews were completed once study team members agreed that no new information was being shared by interviewees, initial codes were repeated, and thematic saturation had been reached based on review of the interview transcripts.

### Reflexivity and trustworthiness

Interviews were completed by two PhD prepared study team members who are both faculty members and have training and experience conducting semi-structured interviews. The study team has completed multiple studies assessing similar research questions and have expertise in hospital community benefit services, community health, and equity. Based on prior quantitative research results, which assessed characteristics of hospitals that consider equity in their community health needs assessments, the study team was interested in gaining more in-depth insight through the current qualitative study. The investigators had no relationships with the interviewees and assumed that interviewees might be hesitant to answer questions that seemed accusatory or overtly critical of their organizations’ equity and community engaged practices. The study team was careful to construct neutral, open ended questions that would allow interviewees to fully express their experiences and thoughts. The interview guide was piloted with two community benefit leaders to ensure it would be perceived as relevant and neutral.

Member checking was done during and at the end of interviews to ensure that the study team correctly understood the interviewees’ statements and point of view. At the end of each interview, participants were asked if there was any additional information they wanted to share with the study team and provided the opportunity to ask any questions at that time or anytime afterwards via email or phone. To ensure participants’ words were accurately captured, interviews were transcribed verbatim.

### Data analysis

Interviews were recorded, transcribed verbatim, and coded by two members of the research team using Dedoose software (v. 9.2.004). Interview transcripts were analyzed, and data were coded and then ascribed to themes using directed content analysis for *a priori* thematic coding. Themes were developed a priori from the five racial equity strategies in the conceptual model. First, interviews were read for any words or phrases (i.e., race, minority, color, ethnicity, equity, disadvantaged/disinvested) related to the study’s conceptual model (EquIR and Browne’s Racial Equity approach). Related words and phrases were coded and then re-read and collated into themes according to Browne’s Racial Equity strategies (i.e., ‘prioritizing community building strategies in communities impacted most by racism) to gain insight into whether or not and how racial equity was prioritized. One member of the study team completed initial coding and developed the codebook. A second team member coded 20% of interviews independently. Team members met to discuss findings and any discrepancies in coding were resolved.

## Results

### Participant characteristics

Twenty-four interviews were held with leaders of 23 different hospitals and health systems representing the Northeast, South, Midwest and West US regions (Table 1). All participants who agreed to be interviewed completed one interview which lasted between 30 and 80 min. Fourteen of the organizations represented were teaching hospitals and eighteen were members of health systems. Fifteen had fewer than 500 beds, with hospital size ranging from 71 to 1,267 total beds. Only three of the organizations were located outside of urban areas and all except one were located in Medicaid expansion states. Fourteen were in states with community benefit policies or requirements. The average revenue was 1.5 billion dollars, with a range of 2 million to 7.4 billion dollars.

**Table 1:** Description of interviewee positions and represented healthcare organizations.

	Title of interviewee	Rurality	Total revenue (Millions USD)	Teaching	Beds	Health system	Medicaid expansion state	State requirement
INT1	Director of Innovations in Healthier Communities	1	4,346	Y	1,135	Y	Y	N
INT2	Community Health Supervisor	1	343	Y	230	Y	Y	N
INT3	Community Benefit Coordinator	1	12	Y	874	Y	Y	Y
INT4	VP Of Community Health and Well Being	1	2,599	Y	395	Y	Y	N
INT5	Community Outreach Director	1	1,470	Y	1,000	Y	Y	Y
INT6	Administrative Fellow	1	410	Y	325	Y	Y	N
INT7	VP of Community Health	1	2,042	Y	395	N	Y	N
INT8	Manager of Community Benefits	1	1,617	N	71	Y	Y	N
INT9	Senior Director, Government and Community Relations	1	7,032	Y	1,267	Y	Y	Y
INT10	Director of Community Impact in SDOH	1	274	N	794	Y	Y	Y
INT11	Director of Community Benefits	1	924	Y	625	Y	Y	N
INT12	Health System Director for CHNA and Community Benefit	1	2	Y	615	Y	Y	Y
INT13	Quality Program Coordinator	10	54	N	25	Y	Y	Y
INT14	Director of Population Health Programs and Strategies	7	117	N	70	Y	Y	Y
INT15	Community Impact Coordinator	4	380	N	301	N	Y	Y
INT16	Clinical Nutrition Manager	1	865	Y	481	N	Y	Y
INT17	Vice President of Healthy Communities	1	751	N	301	N	Y	Y
INT18	Community Benefit and Mission Outreach	1	374	N	347	N	Y	N
INT19	Director of Missions	1	158	Y	1,005	Y	Y	N
INT20	Community Relations Coordinator	1	546	Y	230	Y	Y	N
INT21	System Director of Community Health Impact	1	63	N	208	Y	Y	Y
INT22	Director of Population and Community Health	1	266	N	239	Y	Y	Y
INT23	Coordinator of Community Grants	1	4,346	Y	1,135	Y	Y	N
INT24	Director of Missions	1	7,390	Y	356	Y	N	Y

Rurality code: 1 = Metropolitan area core; 4 = Micropolitan area core: primary flow within an urban cluster of 10,000–49,999; 7 = Small town core: primary flow within an urban cluster of 2,500 to 9,999; 10 = Rural areas.

### Racial equity approaches to community benefit initiatives designed to address social and structural determinants of health

The racial equity approach to community benefit (as described above) includes five strategies. Two of the 23 health systems we interviewed mentioned engaging in all five strategies of the racial equity approach. Three systems discussed using one strategy, four systems mentioned using two, four systems used three strategies, and three systems used four strategies. Eight did not mention any of the five race-based strategies. The strategies most likely to be mentioned by interviewees were (1) prioritizing communities impacted by racism, (2) allocating resources along racial lines, and (3) providing leadership and employment opportunities. The strategies least likely to be mentioned were (1) identifying and addressing intersecting identities and (2) removing barriers to allocating resources (Table 2).

**Table 2:** Most and least used racial equity strategies and representative quotes.

Most used racial equity strategies	
Prioritizing communities impacted by racism	“We’ve really looked at very broadly how we need to invest in the social determinants of health and those conditions that lead to differences in our health outcomes. I think what we have not done to date… is really the equity component. The ways in which we are understanding which populations need more investment…can we invest upstream further…Is there an advocacy component, is there more of a systemic or a structural investment we can make?” *-Int12*
Allocating resources along racial lines	One system discussed the importance of allocating resources along racial lines, and following up to ensure the intended populations was being reached: “…we are doing good data collection as we move through offering the program or initiative to be able to then really look at, who are we serving? Is this making an impact in that intended audience that we set out to assist in some way? ”*-Int11*
Providing leadership and employment opportunities	“So, you know, high incidence of infant mortality…take the service to the people where they are, support those trusted nonprofits who are capable and can do the work, but can do it better if they have the support of an anchor institution like us, so that’s something that we are currently involved with. Our talent acquisition team from HR works with this population as well to make them available of job opportunities [here] because economic empowerment is a part of that as well.” *-Int9*
Least used racial equity strategies	
Identifying and addressing intersecting identities	“So, we did this exercise of prioritizing zip codes across our system. High poverty, high Black, LatinX populations. Focus on that intersection of zip codes where you do your interventions, where you find your partners, where you develop relationships.” *-Int4*
Removing barriers to allocating resources along racial lines	Our purchasing practices, sometimes they get a little bit challenging because we were part of a larger [existing purchasing agreement]. Yet our vice president of procurement attends our local chamber [phone] calls about equitable purchasing and such. And so we are finding local ways that we can look at minority women that are in small owned businesses to do work with and for us, …, depending upon what the need or the issue is, those come to our community benefit work group.” *-Int19*

#### Strategy 1: prioritizing communities impacted by racism

A racial equity approach begins with identifying and implementing programs and policies that prioritize communities disadvantaged due to economic and resource disinvestment and racism. Twenty of the 23 interviewees stated that their hospitals have started disaggregating data based on sociodemographic characteristics like race, zip code, ethnicity, and sexuality, which can help elucidate and quantify inequities. Zip code was mentioned most often.

“ … they have to look at a breakdown of data of persons served based on race, ethnicity, and gender … insurance, and then also their social determinants of health - it is critical to have that data … so they can be focused on how their county is improving…they could look at various zip codes that have high levels of food deserts.” -*Int4*

Though the majority of organizations collected data on race and ethnicity, only about half (*n* = 11) of the interviewees discussed strategies aimed at developing programs or policies that prioritized historically marginalized communities based specifically on racial equity.

Interviewees provided examples of programs and organizational processes they used to focus on racial equity. For example, one health system discussed partnering with a community organization to focus specifically on providing educational opportunities for impoverished children from a historically marginalized ethnic group in their area. “The [community organization] was specifically to help orphans and children. So a lot of their youth are exiting the foster system and are all members of the same ethnic group. And so we support the youth from the homeless program, the youths from that group.” *-Int8*.

Another interviewee described the organizational process or approach which they use in departmental and leadership meetings to ensure they are focusing on prioritizing marginalized communities along racial lines. As she stated, they ask themselves a series of questions:

“Have we built pipelines into disinvested communities? Are we lifting people up and into meaningful work?…We established …what we called a community benefit work group to take a look at the sponsorship and donation requests that were coming in from the community to make sure that they were aligned with our strategic priorities and our service lines.” -*Int19*

One of the biggest challenges interviewees discussed was simply bringing up the sensitive subject of race and racism. For some organizations, it was easier to focus on poverty instead of race. They explained that poverty was a less controversial topic and the issue of racism could sometimes be circumvented given that poor communities, particularly in urban areas, were also largely non-White communities. The other consideration, as described by one interviewee, was that:

“…racial diversity is not… It's very small in this community. The biggest diversity that we have is based on income and age…because by 65 and older they[residents] most likely have multiple chronic diseases… but they're also struggling with how to afford and access healthcare.” *-Int13*

#### Strategy 2: intentionally focusing on intersecting identities

The second strategy to incorporate a racial equity approach to community benefit is taking into consideration that populations may have more than one identity (e.g., member of minoritized racial and sexual or gender group) that puts them at even greater risk of poor health. Three interviewees discussed identifying and prioritizing populations with multiple intersecting identities that might increase burden of health disparities. Two of the interviewees discussed indigenous populations struggling with poverty. One interviewee discussed impoverished Black or Latinx populations.

The interviewee stated that while focusing on economic status was more palatable to leaders in the organization, he felt it was important to also address racial equity and structural racism specifically. By combining both, he was able to ultimately show the extremely high percentage of poverty concentrated in Black and LatinX communities, over and above what was seen in poor White neighborhoods that were identified just by looking at zip codes. Several interviewees discussed identifying startling disparities in maternal mortality between Black and White mothers and subsequently their systems began to focus on addressing those disparities in poor neighborhoods in particular. Their original intention was not to address those with intersecting identities, but the data led them to that focus.

#### Strategy 3: allocating resources along racial lines

When implementing equity focused programs, a third racial equity strategy includes providing resources to communities identified as having suffered as a result of policies rooted in racism and potentially experiencing continued challenges because of structural racism. Twelve interviewees described how using a racial equity lens dictated how they distributed resources and designed community benefit programs and policies. One described developing an educational program by partnering with an organization that serves homeless youth in the foster system. To ensure access, they wrote into policy that at least one spot for the program would be specifically for a member of a racially marginalized group. Another interviewee described combining community benefit dollars with state money to develop a community health worker program to teach disease self-management to Native American populations. Another interviewee described the process of connecting and speaking directly to leaders not only within the hospital but in the community to design programs and operationalize strategies to prioritize racial groups who were historically marginalized from accessing resources. The interviewee felt that after the state encouraged efforts to collect internal hospital data on race and ethnicity to delineate disparities in treatment and outcomes, they started pushing for similar data to be tracked for community engaged programs.

One interviewee discussed a partnership with the public school system to address behavioral health in schools with fewer resources. There was no intentional discussion within the organization to allocate resources to schools that serve mostly students from historically marginalized racial and ethnic groups. However, the interviewees felt that implementing the programs in those schools resulted in some level of racial equity given that the overall demographics of the public schools in that city were students of color “…who would not necessarily have exposure to behavioral health resources that were not being provided through the schools.” *-Int7*.

#### Strategy 4: removing barriers to allocating resources along racial lines

Not only should a racial equity approach include strategies to allocate resources to communities impacted by racism, but also strategies to remove barriers that may prevent or make it difficult for those communities to access and utilize those resources. This step was identified by interviewees as one of the more challenging pieces of the racial equity approach. “…It took us like 4 years to figure out how to buy more from minority women owned businesses, maybe five, and the same with hiring…but like we have been able to move, like it’s a very like tangible thing” *-Int6*.

Overall, seven interviewees discussed why it was so challenging to remove barriers and some of their successes in overcoming challenges. When trying to support minority owned businesses and vendors, common issues identified were (1) health systems not knowing how to adequately make minority or women owned or serving organizations aware of opportunities to partner, (2) minority businesses not having the capacity – experience, human capital or resources - to compete for or complete larger projects, or (3) existing purchasing agreements with large organizations that shut out locally operated minority owned or serving organizations. One interviewee described how their organization made an effort to attend local community business events to meet those challenges (Table 2). Another hospital’s strategy for working with new minority owned businesses that might have less experience, is to provide additional grant writing support.

“So if it's a newer organization or someone who doesn't have a lot of experience writing grants, we provide $1,500 to them to hire a grant writer, to do focus groups, to work on different things in order to give them a little leg up, so to speak. We don't want folks to feel like that they are being left behind because they don't have a grant writer on their staff. ”-*Int15*

A final impediment to allocating resources along racial lines that interviewees emphasized was simply bringing up the topics of race and racism within their organizations. As one interviewee stated when talking about their organization’s approach to equity,” I think it was a type of equity, like low-income, socio-economic…[but] people get real uncomfortable when you start talking about racism and race and equity, and I think that’s something that we continue to work through”*(Int10)*. Beyond difficulties discussing race, interviewees stated that some in the organization had concerns about what they perceived as not treating all populations they served equally. They needed to educate colleagues in order to offer a sound rationale for distributing resources differently and creating a cultural shift in thinking. As one leader described how he explained it to employees:

“I use this thing where we're just sprinkling money around town, it's like, ‘do you want a shower, or do you want sprinkles?’ And sprinkles go away…They will never do anything. It's a drop, and it leaves…we want to shower these communities with resources…with the hopes that the shower actually provides enough resources and enough momentum to add more resources. Because once one entity provides a significant amount of resources, it fuels the fire for more…So, [when we] demonstrate that more in fewer places is better” *-Int4*

#### Strategy 5: providing leadership and employment opportunities

The final racial equity strategy is providing leadership and employment opportunities for communities or members of populations that have historically been the target of racism. These opportunities might be within the health system or as part of community-based organizations or coalitions to address community health improvement. Twelve of the interviewees discussed how their health systems addressed this component.

The most commonly cited strategy was developing educational opportunities for youth or other community members to enter into the health professions. This was considered an extremely viable approach because the community’s need for educational and employment opportunities aligns with the hospital’s need to hire a sustainable population of employees. Hiring locally was considered not only a way to ensure a loyal workforce but also a way to ensure the workforce represents the communities that they provide healthcare for, and also a path to ultimately increase wealth in these less resourced communities. Interviewees talked about mentoring programs that allowed them to identify people with an “…interest in healthcare careers and work with people to get them what they need to serve at their highest levels of interest and ability” (*Int 3*). Another hospital works within their walls to offer similar opportunities to employees:

“So how do we partner with our economic development folks and our school tech programs to…help us go from the community health worker to the life coach to the behavioral health social worker, right?…We're doing that with LNAs[nursing assistants] right now… Anybody who's employed [here] can go through an LNA track. So you can basically take a class, become an LNA from an LNA, there's going to be opportunities to become an RN.” *-Int14*

Another system provides opportunities by partnering with and providing resources to local community organizations, trusting their expertise and leadership to address race-based inequities in infant and maternal mortality (Table 2). To further cement their commitment, one system stated that they are a member of a nonprofit dedicated to equity in health. As part of their membership, their leadership agreed that they should sign an anti-racism pledge.

“…[we] signed on to the Healthcare Anchor Network (19) that racism is a public health issue and we signed onto that effort and initiative and we've made the same commitments as far as looking at our hiring practices and who works with us and do we have equitable and just hiring practices.” *-Int19*

One important consideration and challenge, however, was that hospitals’ programs to improve employment opportunities could not be counted as official community benefit for the purpose of tax exemption if they were designed by the hospital specifically to recruit community members to participate in their program with the ultimate goal of employment at that hospital.

## Discussion

### A racial equity approach to community benefit implementation

As part of their efforts to address social needs and social determinants of health, nonprofit hospitals are planning, designing and implementing equity focused programs. Yet there is wide variation across health systems in the extent to which these programs address racial equity and support populations and communities damaged from long existing effects of structural racism. Interviews of 24 healthcare leaders representing nonprofit hospitals across the United States showed that 16 of the health systems represented used at least one racial equity strategy to plan, design or implement community benefit initiatives. Two of the systems provided examples or discussed how they were incorporating all five steps of a racial equity approach to their equity focused community benefit programs.

### Racial equity strategies used while planning and designing community benefit programs – prioritizing affected groups and considering multiple identities

Collecting and disaggregating hospital and community data was an essential element to planning how to prioritize communities affected by racism. Though 20 of the 24 interviewees stated their health systems disaggregated data along racial lines, only 16 mentioned developing and implementing programs that prioritize communities based on race specifically. The gap between recording racial inequities and leveraging that data to strategically design and implement programs may be due to a number of factors. First, hospitals may be disaggregating for the purpose of filling new requirements by national accrediting and governing organizations (e.g., Centers for Medicare & Medicaid Services, Joint Commission on Accreditation of Healthcare Organizations), but are unclear about how to use the data. Second, some hospitals and health systems serve communities with very little racial variability. Third, organizations may have difficulties discussing race, or feel that it is unfair to focus or prioritize communities based on race as they fear it may privilege one group over others in need. Inability to discuss race and racism can affect how systems design programs and allocate resources. In these cases, systems may default to a colorblind focus on poverty, understanding that there may be enough of an overlap between poverty-stricken communities and racial marginalization to compensate for their race neutral stance.

Despite the challenges, many health systems incorporate racial equity approaches into their community benefit initiatives. An important factor that allows systems to move from collecting data to using it to design programs addressing racial equity, are community benefit and health system administrators who can make a clear case to their peers for why focusing on groups affected by racism, especially in combination with other parameters such as poverty, is so important. The American Hospital Association’s Racial Equity Roadmap (adopted from the Massachusetts Department of Health) includes ‘Presenting data in ways that help people make sense of the numbers’ as one of the final steps to ending structural racism (20). Similarly, interviewees also reported that providing information that is specific and relevant to the audience, succinctly and clearly describe the issue that the data exposes and why it is important and describing the historical and structural practices and policies that preceded the race based inequities (20). Another important factor was the commitment by leadership to promote racial equity, for instance in the form of acknowledging that racism is a public health problem and following it up by supporting racial equity focused initiatives in the organization. South, Butler, and Merchant (21) suggest, in addition to ongoing staff education, establishing endowed leadership positions that would be enshrined in the culture and practices of the institutions and lead to sustainable change.

### Racial equity strategies used while implementing community benefit programs – providing leadership and employment opportunities, allocating resources along racial lines, and removing barriers to accessing resources

Interviewees provided concrete examples of initiatives that focus on racial equity. Most of these initiatives involved partnerships between hospitals and trusted community partners such as community colleges and high schools. This strategy was also reported by Ansell et al. (22) when describing one of the most robust examples of hospital driven equity focused community partnerships – West Side United in Chicago. They identified health inequities rooted in structural racism and cultivated cross sector partnerships with community development financial institutions, suppliers, and other health care organizations which prioritize improving health inequities and building wealth among the most affected populations. In our sample, many of the collaborations supported pipeline programs to provide educational, employment, and leadership opportunities for racially marginalized groups. A key to the success of these programs was that the specific needs identified by community partners, or community residents, fulfilled or mirrored the needs identified by the hospital or health system.

Fewer interviewees talked about how specifically they addressed removing barriers to allocating resources along racial lines and providing leadership opportunities. In a study examining nonprofit hospitals’ process for providing equitable community health improvement programs, Rozier and Singh (11) found that less than 10 % of hospitals could describe formal allocation processes for community health improvement budgets. Therefore, it may not be surprising that some may also lack processes to ensure equitable resource allocation. In our sample of health care representatives, those that did develop strategies to increase access to resources emphasized that without using strategies like providing additional administrative support to write grants, or allowing community partners with expertise to lead initiatives, programs would likely not have been successful.

### Implications

Health systems represented in the current study are grappling with whether or not and how to address racial inequity. Similar to previous qualitative studies with hospital leaders (11, 23), most interviewees described health systems that understand the importance of equity. Lessons can be learned from each that shared their experiences to guide suggestions for how organizational level change can aid hospitals and health systems in addressing racial equity.

First, health systems need to communicate with staff and administrators at all levels, from frontline workers to the board, about why they are collecting data on race and how it will be used to improve population health. Various communication methods and ongoing reiteration are important to ensure staff support efforts and will diligently collect necessary data and ensure that senior leaders of the organization and influential department directors begin thinking about and ultimately promoting important racial equity focused initiatives. Internal communications and education should strive to help employees understand their role in combatting disparities, how essential they are, and to give employees a sense of value and importance in achieving the hospitals’ mission. Presentations using data that show disparities across racial and income lines and telling stories about any lives touched through pilot projects, have all been ways of communicating equity-based hospital initiatives. Those approaches can be replicated when discussing how and why the hospital will address structural racism (24).

Second, along the same lines, staff need opportunities to learn and practice how to have uncomfortable conversations about race. Educating staff with factual information about how racism has affected the health and wellness of local communities may be particularly helpful in addition to small discussion groups and trainings that focus on implicit bias and provide space for employees to discuss race and equity in meaningful ways (25). As part of the discussion, health systems should distinguish between race, equity, and poverty and how or why to address them together or separately. In hospital systems where there is contention around discussing race, they may want to focus community benefit efforts solely on underserved zip codes or income inequities initially. Though the most disparate communities may not be prioritized or receive additional support, they may still receive some benefits and this may be the best most immediate solution to begin to address inequity and take a step toward making a small impact on inequities due to structural racism.

Third, although health systems may intend to address racial inequities by providing resources and partnership opportunities to groups most impacted by racism, without providing extra support to ensure small, local, minority owned businesses have the capacity to access resources and opportunities, their efforts may lead to suboptimal results. Reviewing all steps of the planning, design, and implementation process for initiatives like contracting with business and hiring locally may reveal ways to make small process changes to provide better opportunities for marginalized communities to access opportunities that health systems develop.

Finally, health systems should expect that shifting the focus toward racial equity may take a significant amount of time to develop and embed the system wide protocols and community partnerships needed. However, as established anchor institutions in many communities, nonprofit hospitals often already have a comprehensive network of community partners. Thus, they may still be able to leverage those relationships to design small pilot initiatives that are mutually beneficial to both organizations and have the potential to evolve into larger programs that improve racial equity and overall community wellbeing. Limitations.

This study has limitations. First, though gracious in their willingness to participate, interviewees acting as representatives of their institutions may have exercised some degree of constraint in their answers to avoid an inaccurate understanding of their organizations’ actions or revealing information that could be misconstrued and misrepresent the organization’s actions and intentions. We attempted to mitigate this limitation by anonymizing all results or reporting in aggregate, letting participants know the answers were confidential, giving participants the option to not answer any questions they were uncomfortable answering, and reflecting their answers back to them to ensure accurate understanding throughout the interviews.

Another limitation was that hospitals do not have one streamlined definition of racial equity, or universally accepted terms that they use to describe their equity and racial equity activities. For instance, some institutions may discuss structural determinants of health or disinvested communities of color or zip codes, without mentioning racial equity. Therefore, interviewees may describe racial equity focused initiatives without thinking to use that term. The study team attempted to mitigate this by first asking an equity focused question followed by a specific prompt which included the term ‘race’ and an example of a commonly used racial equity strategy (intentional disaggregated data collection along racial lines), and second by analyzing data for ‘racial equity’ focused initiatives by also searching for equivalent terms (i.e., race, minority, color, ethnicity, equity, disadvantaged/disinvested).

## Conclusion

As national organizations expand requirements to address social determinants of health and equity initiatives, more hospitals will need roadmaps for how to include equity approaches that address structural racism in order to impact upstream determinants of health and improve the health of communities they identify as needing more support. Health systems have the potential, partnerships, and resources to incorporate a racial equity lens into the planning, design, and implementation of their community-based initiatives. Utilizing a racial equity approach that includes prioritizing affected communities and providing resources and strategies to overcome barriers to accessing those resources is a template that can be used by hospitals in racially diverse communities to get closer to more effectively achieving this goal.

## Data availability statement

The datasets presented in this article are not readily available because while data is reported in aggregate and statements are anonymized, the raw interview data does contain information that could be traced back to individual participant organizations. Requests to access the datasets should be directed to chconley@umich.edu.
